# Traumatic brain injury in Uganda: exploring the use of a hospital based registry for measuring burden and outcomes

**DOI:** 10.1186/s13104-018-3419-1

**Published:** 2018-05-15

**Authors:** Amber Mehmood, Nukhba Zia, Connie Hoe, Olive Kobusingye, Hussein Ssenyojo, Adnan A. Hyder

**Affiliations:** 1 Johns Hopkins International Injury Research Unit, Health Systems Program, Department of International Health, Johns Hopkins University Bloomberg School of Public Health, Baltimore, MD USA; 2 Makerere University’s School of Public Health, Kampala, Uganda; 3 Department of Neurosurgery, Mulago Hospital, Kampala, Uganda

**Keywords:** Traumatic brain injuries, Registry, Uganda, Injury, Low-income country, Trauma, Surveillance

## Abstract

**Objective:**

Lack of data on traumatic brain injuries (TBI) hinders the appreciation of the true magnitude of the TBI burden. This paper describes a scientific approach for hospital based systematic data collection in a low-income country. The registry is based on the evaluation framework for injury surveillance systems which comprises a four-step approach: (1) identifying characteristics that assess a surveillance system, (2) review of the identified variables based on adopted specific, measurable, assignable, realistic, and time-related criteria, (3) assessment of the proposed variables and system characteristics by an expert panel, and (4) development and application of a rating system.

**Results:**

The electronic hospital-based TBI registry is designed through a collaborative approach to capture comprehensive, yet context specific, information on each TBI case, from the time of injury until death or discharge from the hospital. It includes patients’ demographics, pre-hospital and hospital assessment and care, TBI causes, injury severity, and patient outcomes. The registry in Uganda will open the opportunity to replicate the process in other similar context and contribute to a better understanding of TBI in these settings, and feed into the global agenda of reducing deaths and disabilities from TBI in low-and middle-income countries.

**Electronic supplementary material:**

The online version of this article (10.1186/s13104-018-3419-1) contains supplementary material, which is available to authorized users.

## Introduction

Each year over 10 million people globally suffer traumatic brain injury (TBI) [1], which is a leading cause of brain disorders and disability worldwide. Road traffic injuries (RTI), which account for 60% of TBI cases, ranked as 8th leading cause of death in 2015 (1.2 million deaths globally); an increase of about 19.7% since 1990 [2]. Other important contributors to TBI include falls (20–30%), and violence (10%) [2, 3].

TBI incidence in sub-Saharan Africa (150–170/100,000) is much higher than the global incidence (106/100,000) [3]. Similarly, incidence of intracranial short-term injuries due to RTI and violence in sub-Saharan Africa are 1.47 and 3.34 times higher than global rates respectively. Whereas incidence rates of intracranial long-term injuries due to war, violence and other unintentional injuries are 5.44, 3.37 and 1.86 times higher than their respective global incidence rates. Rapid motorization and conflicts have been attributed as important causes of the relatively higher TBI incidence in the region [4].

As with majority of sub-Saharan countries, data on the burden of injury and TBI in Uganda is scarce. According to a Ministry of Health report, the capital city Kampala had an estimated annual injury incidence of 116/1000, an injury mortality rate of 220/100,000, an incidence of injury leading to disability of 23/1000, and incidence of non-fatal injuries of 2.8/1000 [5]. Facility-based studies from Uganda estimated the cumulative incidence of TBI hospital admissions at 89/100,000, with alarming figures for TBI-related mortality between 45.3 and 75% [4, 6–8].

The number of deaths from RTI in Uganda has doubled from 3059 in 1990 to nearly 7800 in 2015 [2]. With a growth rate of 3.3%, the population of the capital city Kampala, continues to rise, leading to greater numbers of vehicles and pedestrians on roads, and a resultant increase in road injuries and TBI incidence [9–11]. The day commuter population in Kampala is estimated at about 700,000 people, creating a risk pool of over 2 million people each day on the roads. As a result, approximately 9000 crashes are recorded each year in Kampala, which amounts to 25 crashes per day [12].

To better address the growing TBI burden, there are many critical gaps in knowledge that need to be addressed. Lack of good quality data on TBI hinders understanding of the magnitude of the burden and poses a barrier in identifying risks factors, vulnerable groups, and the impact of interventions. This issue was highlighted in a recent hospital-based study on severe TBI cases, that called for a need for systematic and efficient data collection that could have a positive impact on improving patient outcomes [4]. Thus, the overall goal of this paper is to introduce the development of an internet-based TBI registry in Uganda, based on an injury surveillance framework. The paper describes steps and components of the registry, Kampala internet-based Traumatic Brain Injury Registry (KiTBIR), which is the first of its kind in Uganda to understand the hospital-based TBI burden, risk factors in different populations and age groups, documentation of care processes, and indicators for quality of care. It is expected that the evidence generated from KiTBIR will facilitate the development of TBI management guidelines in resource-constraint settings.

## Main text

The registry development was guided by the core principles of injury surveillance, and hospital-based context. The evaluation framework for injury surveillance systems (EFISS) presented by Mitchell et al. [13] describes the scope of the surveillance system in a four-step approach: (1) identifying characteristics that assess a surveillance system, (2) a thorough review of identified characteristics/variables based on adapted specific measurable assignable realistic time-related (SMART) criteria, (3) assessment of the proposed variables and system characteristics by an expert panel, and (4) development and application of a rating system [13].

According to EFISS, injury surveillance system should be designed so that it could be evaluated for its data quality, operational characteristics, and practical considerations [13]. These attributes are summarized in Table 1. This paper focuses on the development and implementation of KiTBIR, where EFISS characteristics were adopted from the planning to the development phase of the registry, to be able to systematically monitor and evaluate specific attributes after implementation. Subsequent papers will discuss KiTBIR variables, results, sustainability and scale-up of the registry to other settings.

**Table 1:** Modified EFISS framework for traumatic brain injury registry in Uganda

	EFISS characteristics	Application to TBI registry (Uganda)
Data quality characteristics		
Data completeness	Data completeness will refer to an assessment of the proportion of: (i) missing; (ii) ‘not known’; (iii) ‘other specified’; and (iv) ‘unspecified’ data recorded for key characteristics of the injured population	This is ensured by developing a set of required questions which have mandatory data entry check points in the electronic version
Sensitivity	Sensitivity will refer to the ability to correctly detect all cases of true injury events that the data collection intended to detect in the target population	The registry allows a “tag” for patients with “suspected TBI”. They are then followed to assess how many are definitive cases of TBI
Specificity	Specificity will refer to the ability to correctly detect all non-injury cases that the data collection should not have detected as injury cases in the target population	The registry allows a “tag” for patients with “suspected TBI”. They are then followed to assess how many are *not* TBI cases
Positive predictive value	The PPV will refer to the number of correctly identified true injury cases divided by the total number of cases that are identified (correctly and incorrectly) as an injury case from the target population	This gives denominator for calculating the proportion of definitive TBI cases among all (definitive and suspected) TBI cases
Representativeness	Representativeness will refer to the ability of the collection to provide an accurate representation of the distribution of key characteristics of the injured population	The registry is piloted in a tertiary care hospital which is the major referral center in Uganda. It will help to capture a representative sample of TBI cases
Operational characteristics		
Clear purpose and objective(s)	The purpose of the injury surveillance system, the reason why the system exists, and objectives of the injury surveillance system, what the information from the system is used for, should be described	The purpose and objectives of the TBI registry is outlined at the beginning of registry implementation and is shared with all the members of local research team
Data collection process	The method of data collection for an injury surveillance system and the number of steps involved in data collection should be examined using a data collection flow chart	A patient flow chart highlighting various steps in patient enrollment and follow-up has been developed and serves as a reference for the data collection team to ensure uniformity in the process. (Additional file 1: patient and data flow for KiTBIR)
Clear case definition	The injury case definition adopted by an injury surveillance system to identify cases should be described	TBI will be defined based on the history of direct injury to head, or a mechanism involving injuries to multiple body regions such as fall or road traffic injuries or assault. Patients with no mechanism suggestive to indicate head injury or traumatic brain injury will not be included
Timeliness	Timeliness will refer to the time taken to accomplish each of the three surveillance phases of: (i) data collection; (ii) data analysis and interpretation; and (iii) dissemination	Regular meetings of the teams ensure the timeliness of each step. A dissemination plan for sharing the results will be developed
Quality control measures	The quality control measures regularly utilized by the agency responsible for the injury surveillance system should be identified	Electronic quality assurance by standardized terms with minimal text entry. Quality control measures include periodic quality checks on database and on-site checks with data collectors
Data confidentiality	The methods by which an individual’s information in the injury surveillance system is safe guarded against disclosure should be described	Personal identifiers are not stored on the database. Additionally, data security measures are taken to ensure data safety on tablets and server during data upload and transfer
System security	The data access requirements (e.g. password protection) that safe guard against the disclosure of confidential information should be described	All tablets that are used for data collection, server and data transfer portal have multiple layers of security to keep data secure at all stages
Uniform classification systems	The classification system(s) used to record information in the injury surveillance system for variables in the WHO’s core minimum and optimal data sets for injury surveillance should be identified	A training manual with standard definitions and details of each variable is available
Practical characteristics		
Data accessibility	The method by which potential data users access data from the injury surveillance system should be reported	The data will be accessible only to the research team. The data will be kept on a secure server. Secure data transfer mechanisms will be established
Routine data analysis	The routine data analyses conducted using data from the injury surveillance system by the agency responsible for the surveillance system should be described	Reports will be generated and detailed data analysis will be conducted every 2 months to explore missing values and to understand emerging trends in the data being collected
Guidance material to aid interpretation	The availability of guidance material on the interpretation of data from the injury surveillance system should be described	A training manual is developed giving information related to each question and its options
Usefulness	Usefulness will refer to the ability to contribute to the identification of potential key areas for preventive action	It is anticipated that this TBI data will help to understand the TBI burden and associated risk factors in Uganda. This evidence will inform the development of clinical guidelines for TBI management in resource constraint settings. This will also facilitate the understanding of feasibility and utility of electronic registry in such settings

**Fig. 1:**
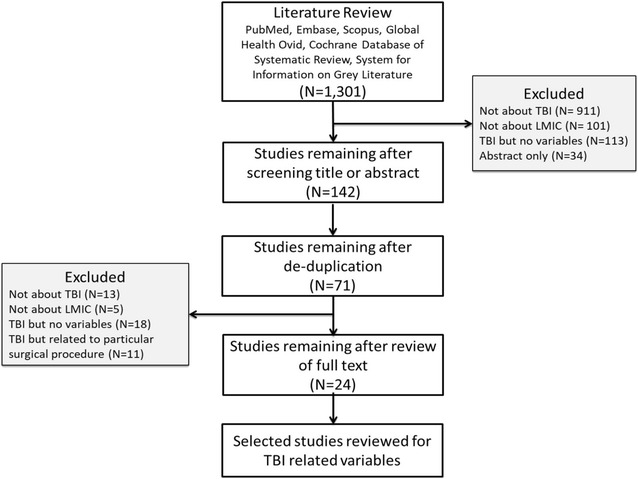
Summary of literature review for identification of core variable for the traumatic brain injury registry

A multi-pronged approach was sought for finalizing the content and scope of KiTBIR. The approach involved a comprehensive literature review, identification of core variables, expert consensus meeting to shortlist the variables according to the scope and case definition. These steps are described in detail below:

The identification of potential variables in KiTBIR involved three strategies: (1) Literature review using search terms and MESH headings for “Brain injury AND Africa” and “Brain injury AND registry AND low-and-middle-income countries (LMICs)”. Several databases were searched using these terms including PubMed/Medline, EMBASE, Scopus, Cochrane Reviews, System for Information on Grey Literature, and Global Health Ovid. The purpose of this review was to understand the published literature on characteristics and risk factors in the context of LMIC and sub-Saharan Africa between year 2006 and 2015 (Fig. 1). Twenty-four papers were selected and independently reviewed by two members of the research team. The result was development of a candidate list of TBI variables; (2) United States National Institute of Neurological Disorders and Stroke (NINDS) guidelines were reviewed for case definitions, TBI signs and symptoms; and (3) review of the previous work done by the team on development and implementation of injury surveillance tools and trauma registries [14, 15].

Core variables covered three main areas: risk factors, clinical care, and patient outcomes. These variables provide context-specific details of hospital burden of TBI, care processes and pre-hospital, emergency department (ED) and inpatient interventions. In addition, date/time variables for injury event, hospital presentation, triage and disposition were included to understand potential delays in care provision. Outcomes at discharge from the hospital was based on Glasgow Outcome Scale which has five categories; death, persistent vegetative state, severe disability, moderate disability and good recovery [16]. Scoring systems such as Kampala trauma score (KTS) was also proposed to measure the injury severity, and risk-adjusted outcome comparisons [17–19]. The final list of variables is available upon request.

The registry was envisioned to create a foundation for TBI prevention and improving quality of care by embedding indicators and outcomes for injury control, access to hospital care, care processes and outcomes. A multidisciplinary consensus meeting was held to finalize the scope of KiTBIR, core variables, data collection platform, and implementation process. The group consisted of local, regional and international public health practitioners, clinicians including neurosurgeons, injury prevention experts and information technology professionals. Time burden, local healthcare delivery processes, and data collection feasibility was also considered in finalization of the variables. Consensus was developed for the inclusion/exclusion criteria, operational definitions, and key performance indicators. Additionally, approaches to get reliable information, data collection procedures, training needs and ethical considerations were also established.

The final KiTBIR registry consists of six broad sections with 97 variables. Each section covers 5–29 questions, which are illustrated in Table 2 and include patients’ demographics, pre-hospital and hospital assessment and care, external causes of injuries, injury severity measured by KTS and revised trauma score, and patient outcome. KiTBIR provides an opportunity for validation of KTS in TBI population of Uganda.

**Table 2:** Sections and variables in KiTBIR

Sections/domains	Sample variables	References^a^
1. Patient demographic information	Age, sex, area of residence, marital status, highest education, employment status	[4, 23–43]
2. Pre-hospital care	Care details included first aid and pre-hospital assessment, transport time, mode of arrival	[25, 29, 37, 39, 40]
3. Injury event information	Date and time of injury, place and activity of injury	[4, 23–26, 28–43]
4. Emergency room assessment and treatment	Vital signs, GCS, pre-existing conditions, suspicion of alcohol and substance use, lab and radiology investigations, patient management, respiratory support, ED disposition	[4, 23, 25–37, 39, 41]
5. Inpatient care	Surgical treatment, complications, ICU care	[23–25, 32, 34, 37, 39, 42]
6. Discharge	Discharge outcome, length of stay	[4, 23–32, 34–46]
Quality indicators	Duration between injury and arrival, time seen by neurosurgical team, date and time of intubation, ED length of stay, date and time of CT and surgical intervention	
Injury and outcome measures	Revised trauma score, Kampala score, Glasgow outcome score; pre- and post-resuscitation GCS	

^a^Studies that mentioned the listed variables selected for KiTBIR

The registry was digitized using a m-health platform that was developed and pretested according to the local needs and resources available for TBI patients at the tertiary-care hospital in Kampala, Uganda.

In the digital platform, the flow of the information and organization of different sections was based on the usual course of patient in a tertiary-care hospital of Uganda (Additional file 1: Figure S1: patient and data flow for KiTBIR). Patients with TBI typically present to the ED where staff completes initial assessment to assess patient’s condition and injuries, followed by specialty consultation based on the injury severity and need for hospital admission. Patients requiring inpatient admission are moved to neurosurgery ward while patients with less severe injuries are observed and discharged from ED. This patient flow is considered in designing the content and flow of questions in each section of KiTBIR to facilitate data collection.

Several steps were taken to develop a user-friendly and standardized data collection tool. OpenDataKit (https://opendatakit.org), an open source software for mobile data collection, was used and an Aggregate on a cloud server in Uganda was setup. Questionnaires were developed in Microsoft Excel XLSFORM, and uploaded to the Aggregate for data collection. The questionnaires largely consisted of dropdown menus and check boxes, along with mandatory fields such as age, sex, vital signs. The ODK-Collect app was downloaded on the Android tablets, secured through user-specific password, for data collection. Completed data collection forms on the tablet could be uploaded over Wi-Fi or internet connection to the Aggregate on the cloud server. Data quality control measures, confidentiality, and system security were in place. The encryption of the device was supported by elliptic curve cryptography and script; this provided additional security of the data with the ability to remotely activate android device manager to lock or erase information in case of stolen or lost device.

## Strengths and limitations

This is one of the first studies designed to collect prospective hospital-based data on TBI patients in a large tertiary-care hospital of a low-income setting, employing electronic data collection methods. KiTBIR demonstrated several advantages: *First*, it feeds into a surveillance system, documents the hospital-based TBI burden, risk factors, injury mechanisms, outcomes, allows to fill information gaps on the vulnerable populations, and develop strategies for prevention and control [20].

*Second*, KiTBIR documents care processes and performance indicators in a low-resource setting, that functions with limited infrastructure, human resources, access to care, and efficiency compared to high-income countries [21]. Combining core surveillance measures with hospital-based care processes and outcome, makes KiTBIR a robust tool to perform dual function of surveillance and monitoring quality of care. Development and implementation of clinical management protocols and data-driven policies to improve outcomes of TBI patients in Uganda will be the ultimate impact of the registry.

*Third*, this hand-held based data collection tool uses innovative approach which could become a foundation for multicenter injury database [22]. Using open-source software and smart electronic devices is the way forward to streamlining data collection in setups lacking electronic medical record systems. The collaborative approach has resulted in understanding local context and developed local capacity for future sustainability and integration of the registry into the current system at the hospital.

*Lastly,* utilization of the EFISS framework provided the basis of the planning and development of KiTBIR, and ensured that data quality, operational challenges and practical considerations were taken into account. Similar approaches could guide other researchers and clinicians in development of a comprehensive database for injury surveillance and hospital-based registries. The planning and implementation of KiTBIR, based on EFISS provides a proof of concept that low-cost, good quality, reliable injury surveillance systems could be established and help identify targeted strategies to increase the uptake of the system and expand it to other clinical settings.

There are some limitations of this work. *First*, KiTBIR is designed to collect data on TBI patients from their point of entry to exit from the hospital. In a resource-limited tertiary care setting, tracking patients through their entire course, especially those involving multiple departments and locations, might present a unique challenge. There is lack of centralized information system to update patients’ location or discharge in real time, and this could potentially result in missing information, or loss of patient follow-up after leaving ED.

*Second*, since this is the first time that tablets are used for data collection in the hospital, there is a risk of loss of data in case of tablet malfunction, theft, etc., if not timely uploaded on the servers. In such instances, the data will not be retrievable. During implementation phase, effort would be made to ensure data submission within 24 h of patient presentation to the ED.

A customized electronic TBI registry will open opportunity to replicate the process in similar settings and contribute to the global agenda of reducing deaths and disabilities from TBI in low-income countries.

## Additional file

**Additional file 1.** Patient and data flow for KiTBIR.

## Abbreviations

EFISS: evaluation framework for injury surveillance systems
KiTBIR: Kampala internet-based Traumatic Brain Injury Registry
LIC: low-income countries
LMIC: low-and-middle income country
RTI: road traffic injury
SMART: specific measurable assignable realistic time
TBI: traumatic brain injuries
WHO: World Health Organization
