# Supplementary material for: Traumatic brain injury in Uganda: exploring the use of a hospital based registry for measuring burden and outcomes
Source: BMC Res Notes. 2018 May 15;11:299. doi: 10.1186/s13104-018-3419-1 (PMC5952367; doi:10.1186/s13104-018-3419-1)
Supplement: Supplementary file 1 — Additional file 1. Patient and data flow for KiTBIR. [file 13104_2018_3419_MOESM1_ESM.docx]

**Additional file 1: Figure S1 Patient and data flow for KiTBIR**
